# The Effect of Ghrelin upon the Early Immune Response in Lean and Obese Mice during Sepsis

**DOI:** 10.1371/journal.pone.0122211

**Published:** 2015-04-06

**Authors:** Daniel Siegl, Emily F. Midura, Thorsten Annecke, Peter Conzen, Charles C. Caldwell, Johannes Tschoep

**Affiliations:** 1 Walter-Brendel-Centre of Experimental Medicine, Ludwig-Maximilians-University of Munich, Munich, Germany; 2 Department of Anaesthesiology, University Hospital Munich (LMU), Munich, Germany; 3 Division of Research, Department of Surgery, University of Cincinnati College of Medicine, Cincinnati, Ohio, United States of America; 4 Department of Anaesthesiology and Intensive Care Medicine, University Hospital of Cologne, Cologne, Germany; Hosptial Infantil Universitario Niño Jesús, CIBEROBN, SPAIN

## Abstract

**Introduction:**

It is well established that obesity-related hormones can have modulatory effects associated with the immune response. Ghrelin, a hormone mainly derived from endocrine cells of the gastric mucosa, regulates appetite, energy expenditure and body weight counteracting leptin, a hormone mainly derived from adipocytes. Additionally, receptors of both have been detected on immune cells and demonstrated an immune regulatory function during sepsis.

**Methods:**

In the present study, the effect of peripheral ghrelin administration on early immune response and survival was investigated with lean mice and mice with diet-induced obesity using cecal ligation and puncture to induce sepsis.

**Results:**

In the obese group, we found that ghrelin treatment improved survival, ameliorated hypothermia, and increased hyperleptinemia as compared to the lean controls. We also observed that ghrelin treatment divergently regulated serum IL-1ß and TNF-α concentrations in both lean and obese septic mice. Ghrelin treatment initially decreased but later resulted in increased bacteriaemia in lean mice while having no impact upon obese mice. Similarly, ghrelin treatment increased early neutrophil oxidative burst while causing a decrease 48 hours after sepsis inducement.

**Conclusion:**

In conclusion, as the immune response to sepsis temporally changes, ghrelin treatment differentially mediates this response. Specifically, we observed that ghrelin conferred protective effects during the early phase of sepsis, but during the later phase deteriorated immune response and outcome. These adverse effects were more pronounced upon lean mice as compared to obese mice.

## Introduction

Sepsis is a disease with increasing incidence worldwide. This disease is defined as a systemic inflammatory response syndrome coupled with a documented infection [1]. A dysregulated inflammatory response can result in an initial response dominated by hyper-inflammation followed by persistent immune suppression, multi-organ damage and death [2]. Currently, treatment mainly consists of supportive care with no effective therapy for combating immune dysfunction [3]. Obesity represents a growing health problem, especially in industrialized countries where more than 50% of adults are already overweight or obese [4]. Obesity is a risk factor for comorbidities such as hypertension, cardiovascular diseases and diabetes. Furthermore, obesity can detrimentally influence the immune system [5]. It remains unclear whether obesity-induced changes improve or worsen outcome and the immune response during sepsis [6,7], but there is increasing evidence that low-grade obesity may be protective against sepsis-induced mortality [8,9].

A variety of cytokines, second messengers and hormones coordinate the host immune response to sepsis. Ghrelin is a gut-brain peptide originally derived from the gastric mucosa [10] showing increases of systemic baseline levels upon moderate caloric restriction [11]. Besides its function in regulation of appetite and metabolic balance and its role as an anti-obesity target and counterpart of leptin, it can also modulate leukocyte function. In early sepsis, it has been demonstrated that ghrelin improved organ perfusion and function and supported anti-inflammatory actions [12]. Ghrelin is also known to stimulate the vagus nerve and affects the vascular system [13]. Ghrelin receptors have been detected in cardiovascular tissue and ghrelin treatment can help correct the abnormalities of hemodynamics in rats with septic shock [14]. Interestingly, ghrelin levels during sepsis have been demonstrated to either increase or decrease [15,16]. Chang et al. showed that an additional ghrelin treatment could effectively increase serum ghrelin levels [14].

Leptin, a product of the obese (ob) gene and secreted by adipocytes, was initially observed as satiety factor regulating food intake, body weight and a variety of biological effects by its peripheral and central action [17]. Additionally, leptin receptors were also found on peripheral leukocytes [18]. Leptin has specific effects on T-lymphocytes and monocytes and improves cytokine response and demonstrated survival benefits during sepsis [19,20]. It is unclear whether ghrelin and leptin have antagonistic actions during host immune response during sepsis. Both are found to increase in sepsis, with ghrelin suggested to inhibit leptin-induced cytokine expression [16,21]. In contrast, single endotoxin injections suppressed plasma ghrelin levels in rats [22]. Additionally, it is unclear, whether ghrelin has different effects on the immune response in sepsis in lean and obese conditions.

In the present study, we investigated the influence of exogenous ghrelin administration on the early immune response during sepsis using obese and lean control mice compared to both untreated groups. To simulate an induced low-grade obesity (class 1) as seen in humans, we used a long-term high-fat-diet feeding model (12 weeks) on mice followed by induction of polymicrobial sepsis by cecal ligation and puncture (CLP). The CLP method represents a peritonitis model with clinical features of polymicrobial infection consistent with those of peritonitis in humans [23]. We hypothesized that ghrelin would be protective in this model and improve survival by influencing the cytokine and cellular immune response.

## Materials and Methods

### Ethics Statement

All experiments of this study involving animals were performed according to the guidelines of the local Animal Use and Care Committee and the National Animal Welfare Law and after approval by the local Animal Use and Care Committee (Government of Bavaria, Munich, Germany; Permit Number: 55.2-1-54-2531-47-10) and are in agreement with the guidelines for the proper use of animals in biomedical research and the guidelines of the European Communities Directive 86/609/EEC regulating animal research.

### Animal studies

Male C57Bl/6J (wild-type) mice 7 weeks of age (n = 170) were obtained from Janvier SAS (Le Genest Saint Isle, France) and maintained in individually ventilated cages under a 12h light/12h dark cycle under conditions of controlled temperature (22 ± 2°C) and relative humidity (55 ± 5%).

### Feeding of Mice

Mice were maintained *ad libitum* on water and a high fat diet (hfd) (E15186-34; Ssniff, Soest, Germany) for 12 weeks to induce class 1 obesity. The hfd contained mainly saturated fatty acids and the metabolizable energy was from fat (50.0%), protein (22.0%) and carbohydrate (28.0%). Age-matched C57Bl/6J control mice received water and a control chow diet (CD) ad libitum for 12 weeks with calories provided by fat (11.0%), protein (23.0%) and carbohydrate (65.0%).

### Experimental groups

To evaluate basic data (= before) 5 lean control mice and 5 hfd mice were used. For experiments, mice (n = 160) were randomly divided into 4 groups: untreated lean controls (= controls; n = 40), untreated obese (= hfd; n = 40), ghrelin treated lean controls (= controls + ghrelin; n = 40) and ghrelin treated obese (= hfd + ghrelin; n = 40) and were used for survival studies and experiments at three different time points (6h, 24h and 48h) after CLP.

### Cecal ligation and puncture

Mice (19 weeks old) were inflicted with polymicrobial sepsis induced by CLP as described previously [24]. Briefly, the CLP operations were always performed between 9:00 A.M. and 11:59 A.M. Mice were anesthetized with a single intraperitoneal (ip.) injection of pentobarbital (50 mg/kg body weight (BW)) and buprenorphine (0.3 mg/kg BW). They were allowed to spontaneously breathe room air on an electric heating pad. The skin was shaved and disinfected. After a 1 cm laparotomy was made (midline incision through the linea alba while avoiding injury to the abdominal vasculature), the distal 80% of the cecum was ligated with a 3–0 silk tie (Catgut, Markneukirchen, Germany) and punctured once on the anti-mesenteric side with a 23-gauge needle. A small amount of the bowel contents was extruded through the puncture hole to ensure a full thickness perforation. Care was taken not to obstruct the bowel, and this was tested after the animals’ death. The cecum was replaced in its original location, and the midline incision was closed by two-layer suture with 4–0 silk (Catgut, Markneukirchen, Germany). The animals were resuscitated with 1 ml of sterile saline subcutaneously and kept on a heating blanket for 2 hours. After CLP, mice were observed every 24 hours for 10 days or until termination of the study and monitored for body temperature, BW and signs of distress. Mice fulfilling fixed criteria for termination during the experiments were killed.

### Intraperitoneal Ghrelin administration

Murine ghrelin (Tocris, Bristol, United Kingdom) was used for intraperitoneal administration. Stock solution was prepared according to the protocol in sterile H_2_O and stored at -80°C. After CLP, a ghrelin dosage of 0.5 μg/g BW for each injection (ip.) was performed twice a day starting directly after CLP until the study termination. This ghrelin treatment protocol has been shown not to affect sleep-wake activity or motor activity [25].

### Collection of blood, serum and cell-free peritoneal lavage fluid

Whole blood was collected by sterile, percutaneous cardiac puncture. Blood was used for colony forming units and flow cytometry as described there. For serum collection, blood samples were placed in serum separator tubes (BD Biosciences, San Diego, CA, USA), centrifuged at 1,000 x g for 10 minutes with isolated serum subsequently transferred to a new sterile tube. Peritoneal lavage fluid was harvested from mice after aseptic preparation of the abdominal wall, injection of 9 ml of sterile saline into the peritoneal cavity and aspiration of peritoneal fluid. The lavage was next centrifuged for 10 minutes at 450 x g to collect cells.

### Flow cytometry for surface staining

Single cell suspensions of heparinized blood were saved in ice-cold Hanks buffered salt solution (HBSS). After washing twice with HBSS, cells from blood and peritoneal lavage fluid were prepared using standard procedures. Cells were resuspended in fluorescent- activated cell sorting (FACS) buffer (Phosphate buffered saline with 1% bovine albumin and 0.1% azide). To avoid non-specific binding to mouse Fcγ receptors, cells were blocked with anti-mouse CD16/CD32 (20 min, 1 mg/mL; BD Pharmingen, Franklin Lakes, NJ, USA) and 1% rat serum (Life Technologies, Paislay, UK) added to the FACS buffer. Cells were stained in a three-color configuration with Fluorescein (FITC)-, phycoerythrin (PE)- or Peridinin chlorophyll protein (PerCP)-labeled antibodies. The following antibodies were used: anti-Ly6G (clone: 1A8), anti-CD11b (clone: M1/70), anti-NK1.1 (clone: PK136), anti-γδTCR (clone: UC7-13D5), anti-TCRβ (clone: H57-597) (all BD Biosciences, San Diego, CA, USA). The samples were washed thoroughly with FACS buffer and analyzed using a FACScan flow cytometer and Cell Quest software (BD Biosciences, San Diego, CA, USA). Compensation to avoid fluorescence overlap was performed according to the manufacturer’s protocol. Ten to twenty thousand cells were analyzed from each sample. Mean fluorescence intensity (MFI) was measured by flow cytometry as a marker of cell surface expression of analyzed molecules.

### Neutrophil Oxidative Burst Determination

For oxidative burst (spontaneous and stimulated hydrogen peroxide production), cells collected from blood or peritoneal lavage were first incubated with dihydrorhodamine (DHR; 2.5 x 10^–5^ M) and subsequently stimulated with fMLP (10^–5^ M). During this time, rhodamine is formed upon oxidation of DHR by reactive oxygen species (ROS). This reaction was stopped by putting cells on ice. These cells were labeled with PE anti-mouse Ly-6G (Gr-1) and APC anti-mouse CD11b. Using flow analysis, Gr-1^high^ / CD11b positive cells were identified as granulocytes. Intracellular ROS production was indirectly detected on the FL-1 channel by rhodamine intensity.

### Body weight and body temperature

Body weight was measured using a standard scale. Body temperature was measured rectally using a Greisinger GTH1170 thermometer (Greisinger, Regenstauf, Germany).

### Leptin and cytokine measurement

Leptin, interleukin-6 (IL-6), IL-10, IL-1ß and tumor necrosis factor α (TNFα) levels in the peritoneal fluid and serum were analyzed using enzyme-linked immunosorbent assay (ELISA) kits according to the protocol of the manufacturer (PeproTech, Hamburg, Germany) with a sensitivity of 20 pg/ml, 62 pg/ml, 47 pg/ml, 63 pg/ml and 16 pg/ml respectively.

### Leukocyte counts

Numbers of leukocytes from peritoneal lavage fluid were measured using a Coulter® Ac·T diff Analyser (Beckman Coulter, Brea, CA, USA).

### Colony forming units

Bacterial counts were performed on aseptically harvested blood by cardiac puncture and peritoneal lavage fluid both collected before, 24h and 48h after CLP. Samples were serially diluted in sterile saline and cultured on tryptic soy agar pour plates. Plates were incubated at 37°C for 48h, and colony forming unit counts were performed.

### Statistical Analysis

GraphPad Prism 5.0 (GraphPad Software, San Diego, CA, USA) was used for statistical analyses. Quantitative data are presented as mean ± standard error of the mean (SEM). Survival probabilities were graphically assessed by the Kaplan-Meier method, statistical comparisons were performed using the log-rank test for survival. Data were tested for normal distribution using the Kolmogorov-Smirnov test. The nature of hypothesis testing was two-tailed. For simple comparisons of two groups (hfd and controls) at the starting timepoint to define baseline observations (“before” data) a two-tailed Student-t test for unpaired samples was used. For comparisons of body weight and body temperature between multiple groups, a two-way ANOVA for repeated measurements was used followed by a post hoc Bonferroni test. For comparisons of multiple groups concerning the influence of ghrelin on immune response parameters, a two-way ANOVA for unmatched samples was used followed by analyses between groups or within groups over time using a post hoc Bonferroni test. A p-value of 0.05 or less was considered as statistically significant.

## Results

### High-fat-diet results in Class 1 obesity

To investigate a possible influence of ghrelin on early immune response and survival in sepsis and determine obesity-dependent differences we conducted an animal study on septic mice. To simulate a diet-induced obesity as seen in humans, normal C57Bl/6 mice were fed a high-fat-diet (hfd; 60% fat) for 12 weeks. These mice gained more body weight (BW) within 12 weeks of feeding as compared to the control group with normal standard diet (11% fat) resulting in a significant higher (25%) body weight than the controls (35.8 ± 0.5 g vs. 28.6 ± 0.2 g, p < 0.0001). Human weight gain of 25% would result in a BMI of approximately 30 and therefore, the hfd group is representative of the obese class 1.

### Ghrelin treatment reduced short-time survival in sepsis

**Fig 1:**
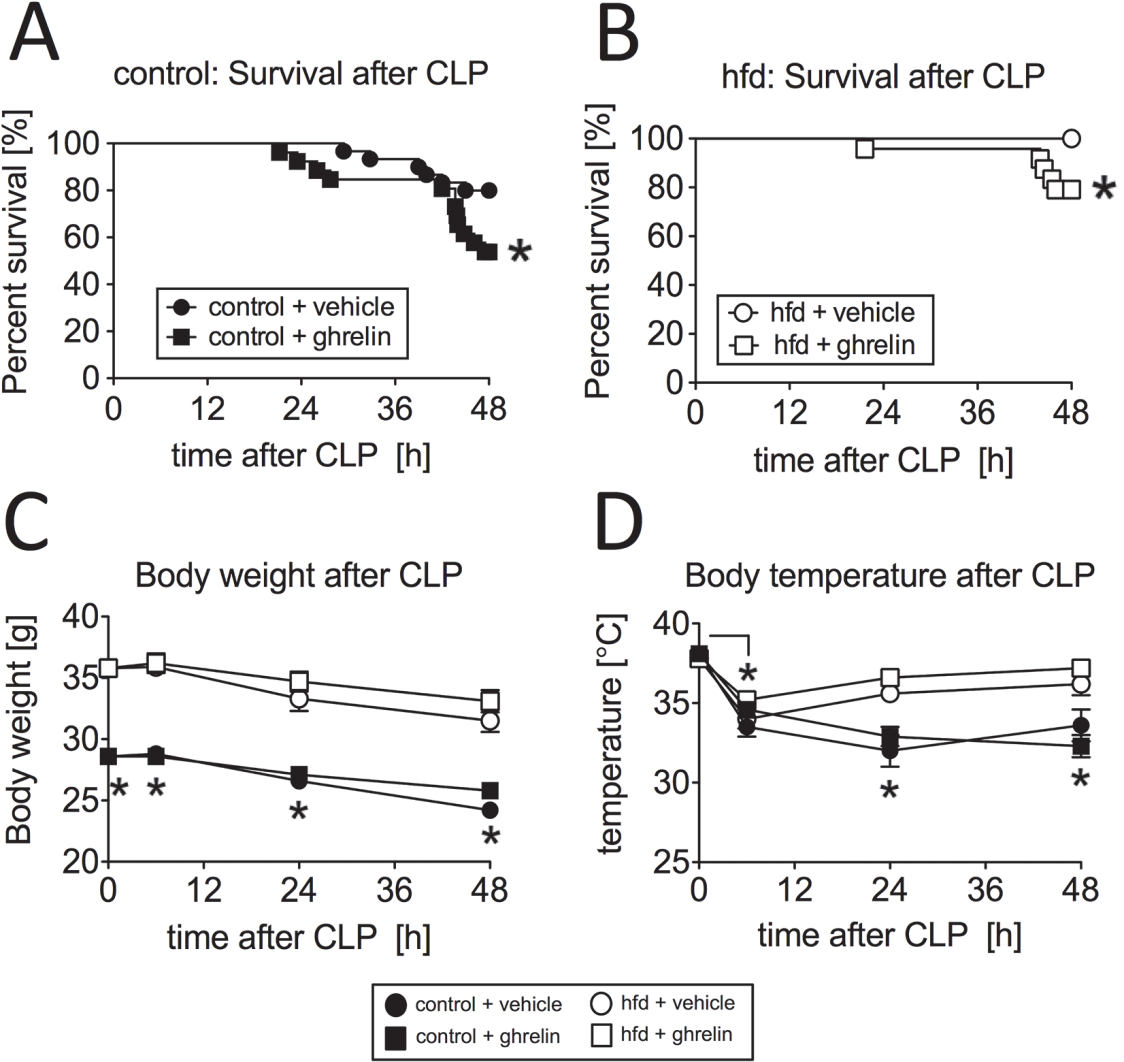
Influence of ghrelin treatment (0.5 μg/g BW, twice a day, intraperitoneal (ip.)) on survival, body weight and body temperature within 48 hours after cecal ligation and puncture (CLP)-induced sepsis. Survival of (A) control mice with ghrelin treatment (ip.) (n = 26) or vehicle (volume adapted, ip.) (n = 30) and (B) mice with chronic high fat diet (hfd) and ghrelin treatment (ip.) (n = 24) or vehicle (volume adapted, ip.) (n = 24) within the first 48 hours after CLP. The survival curve combines data from eight independent experiments; * p < 0.05 of log-rank test as compared to the vehicle treated group. (C) Body weight was measured in control mice (ghrelin: n = 14–40/timepoint, vehicle: n = 11–24/timepoint) and hfd mice (ghrelin: n = 19–38/timepoint, vehicle: n = 13–30/timepoint) before, 6h, 24h and 48h after conducting CLP (combined data from several independent experiments); * p < 0.05 of two-way ANOVA for repeated measurements followed by a *post hoc* Bonferroni test. (D) Body temperature as independent predictor of mortality was measured rectally in control mice (ghrelin: n = 14–40/timepoint, vehicle: n = 11–24/timepoint) and hfd mice (ghrelin: n = 19–38/timepoint, vehicle: n = 13–30/timepoint) before, 6h, 24h and 48h after conducting CLP (combined data from several independent experiments); * p < 0.05 of two-way ANOVA for repeated measurements followed by a *post hoc* Bonferroni test. All values are expressed as means ± SEM.

As we have shown in previous studies, the first 48 hours after CLP-induced sepsis are of particular interest and important for early regulatory mechanisms being responsible for outcome and the course of the immune response [19,20,26,27]. For that reason we focused on this early timeframe. Septic control mice (n = 30) showed a survival rate of 80% within the first 48h after CLP (Fig 1A). Ghrelin treatment (0.5 μg/g BW; twice daily, ip.) starting directly after CLP significantly decreased the survival to 53.8% (p = 0.045). In the septic hfd group we found a 100% survival within the first 48h after CLP (Fig 1B), while ghrelin treatment significantly reduced survival to 79.1% (p = 0.019). Interestingly, survival of the ghrelin treated hfd group was significantly higher (p = 0.042) as compared to the lean cohort treated with ghrelin. There were no differences in survival in both untreated groups (hfd and controls) between injection of vehicle or no injections after CLP (data not shown).

### Effect of ghrelin and obesity on body temperature and weight changes during sepsis

Although there was a sepsis-induced reduction of body weight in both hfd (31.5 ± 0.9 g) and controls (24.2 ± 0.4 g) within the first 48h after CLP, the hfd group maintained a significantly higher body weight (p < 0.0001) as compared to the lean control mice (Fig 1C). Ghrelin treatment had no significant effect on body weight of both groups within the first 48h after CLP (33.0 ± 0.8 g and 25.8 ± 0.5 g). In contrast, body temperature significantly decreased (p < 0.0001) in both groups 6h after CLP starting from 37.8 ± 0.2°C in the obese cohort and 38.0 ± 0.1°C in the lean cohort to 34.0 ± 0.6°C and 33.5 ± 0.6°C, respectively (Fig 1D). While the control group stayed at this lower body temperature at the 24h (32.0 ± 1.0°C) and 48h timepoint (33.6 ± 1.0°C), the temperature of the obese mice significantly increased (35.6 ± 0.5°C and 35.6 ± 0.7°C) and was significantly higher when compared to the control group at these time points (p = 0.0015 and p = 0.043). Ghrelin treatment had no significant influence on body temperature after CLP in control or hfd mice. This is also reflected by the changes of body temperature between the single observed time points. The control group developed a larger decline in temperature as compared to the hfd group at 6h (hfd: -4.2 ± 0.6°C vs. control: -3.3 ± 0.6°C, p = 0.044), 24h (hfd: -1.1 ± 0.7°C vs. control: -4.0 ± 1.2°C, p = 0.040) and 48h (hfd: -0.1 ± 0.3°C vs. control: -2.9 ± 0.3°C, p = 0.0007) after CLP.

### Ghrelin treatment alters levels of early mediators in sepsis

**Fig 2:**
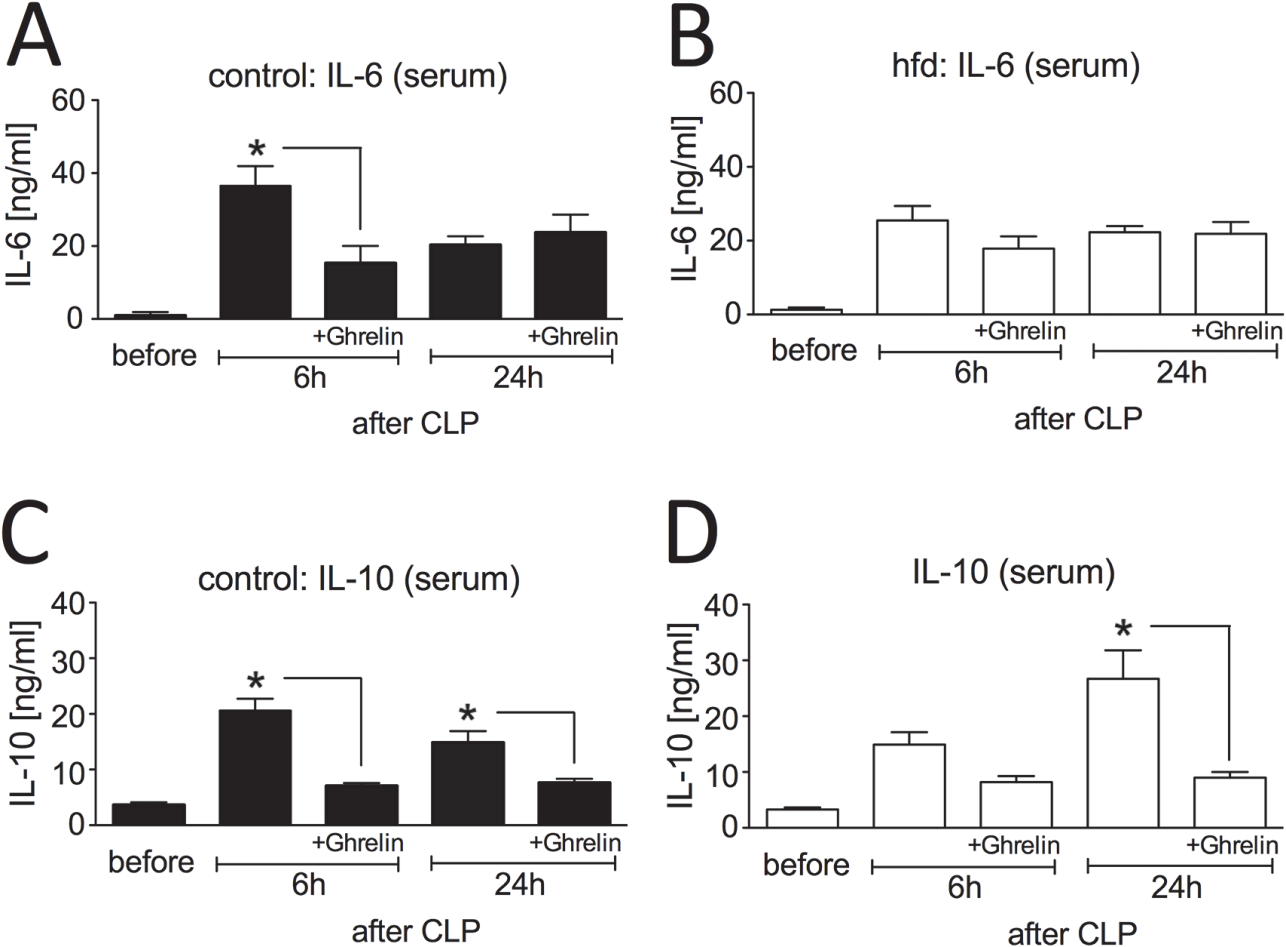
Influence of ghrelin treatment (0.5 μg/g BW, twice a day, intraperitoneal (ip.)) on early cytokine response after cecal ligation and puncture (CLP)-induced sepsis. Serum was collected from control mice (ghrelin: n = 5–9 / timepoint, untreated: n = 4–5 / timepoint) and high fat diet (hfd) mice (ghrelin: n = 4–10 / timepoint, untreated: n = 4–5 / timepoint) before, 6h and 24h following CLP. Serum Interleukin (IL)-6 levels of (A) control and (B) hfd mice and serum IL-10 levels of (C) control and (D) hfd mice were determined by ELISA (combined data from several independent experiments); * indicates p < 0.05 using two-way ANOVA for unmatched samples followed by a *post hoc* Bonferroni test for analyses between more than two groups or within groups over time. All values are expressed as means ± SEM.

We next examined possible influences of ghrelin treatment on key cytokines of the inflammatory response. Serum IL-6 levels are known as a predictor of outcome in sepsis [28]. In both untreated control and in the hfd cohort, a significant increase in IL-6 levels was observed 6 hours after CLP compared to the sham-CLP cohort (control: 36.5 ± 5.4 ng/ml, p = 0.0002, hfd: 25.5 ± 3.9 ng/ml, p = 0.0002) (Fig 2A and 2B). Notably, ghrelin treatment ameliorated the early increase of IL-6 at the 6h time point in serum only in the lean cohort (51% decrease, p = 0.029). IL-10 is an important anti-inflammatory cytokine that can temper the immune response during sepsis. Serum IL-10 levels significantly increased in both groups at the 6 hour time point (control: 20.6 ± 2.1 ng/ml, hfd: 14.9 ± 2.2 ng/ml; p < 0.01 compared to sham) (Fig 2C and 2D). After 24 hours, the IL-10 levels in the hfd group further increased 179%. Ghrelin treatment lead to a reduction of serum IL-10 levels in the controls (6 hours: 7.1 ± 0.5 ng/ml, p = 0.016, 24 hours: 7.6 ± 0.7 ng/ml, p = 0.002) and after 24 hours in the hfd group (9.0 ± 1.1 ng/ml; p = 0.0007). Altogether, ghrelin-treatment had a differential impact upon IL-6 and IL-10 levels in the lean and hfd cohorts.

### Influence of ghrelin treatment on inflammatory mediators in sepsis

**Fig 3:**
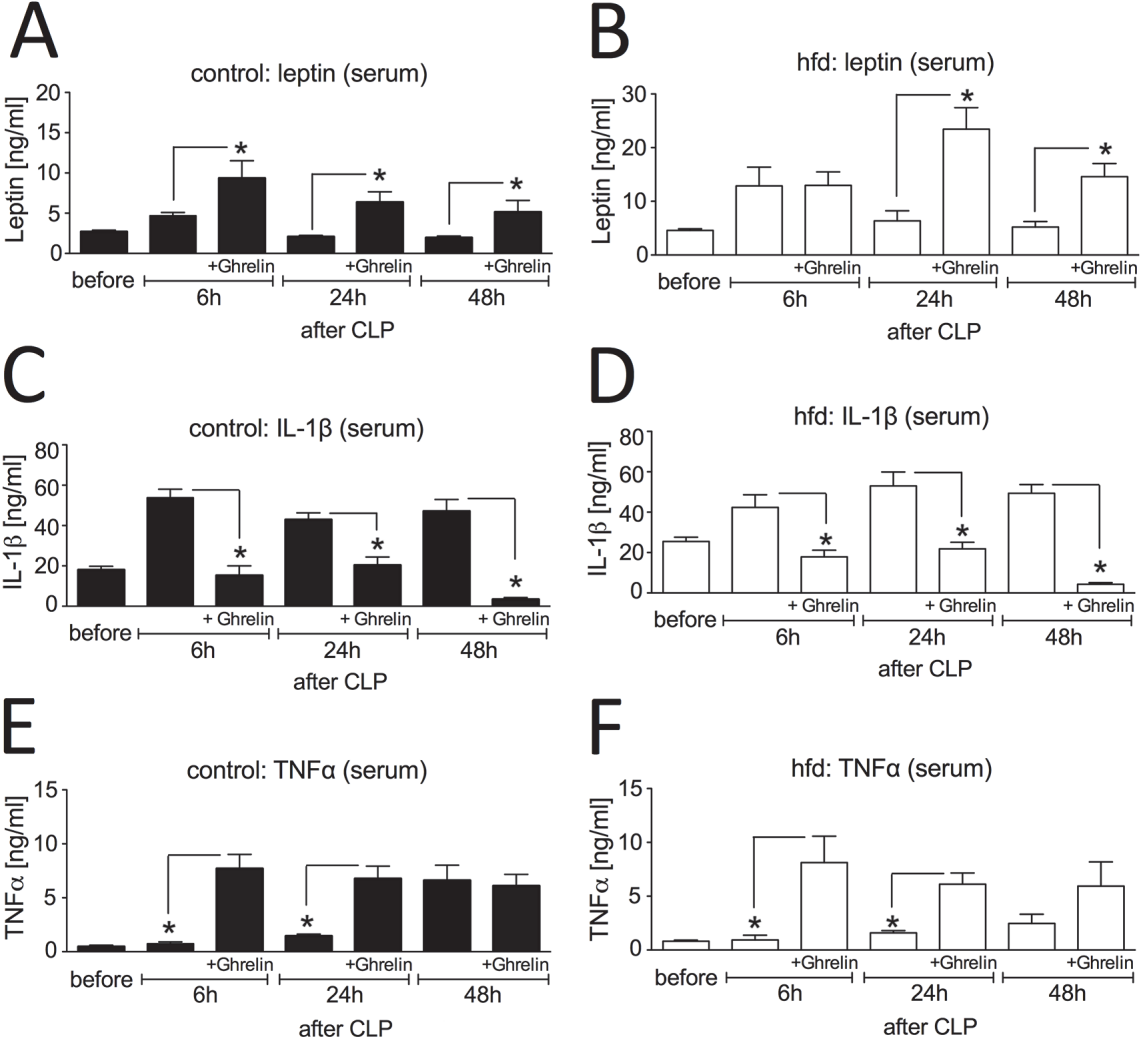
Influence of ghrelin treatment (0.5 μg/g BW, twice a day, intraperitoneal (ip.)) on inflammatory mediators after cecal ligation and puncture (CLP)-induced sepsis. Serum was collected from control mice (ghrelin: n = 5–9/timepoint, untreated: n = 4–5/timepoint) and high fat diet (hfd) mice (ghrelin: n = 4–10/timepoint, untreated: n = 4–5/timepoint) before, 6h, 24h and 48h following CLP. Serum leptin levels of (A) control and (B) hfd mice, Interleukin (IL)-1ß levels of (C) control and (D) hfd mice and tumor necrosis factor (TNF)α levels of (E) control and (F) hfd mice were determined by ELISA (combined data from several independent experiments); * p < 0.05 of two-way ANOVA for unmatched samples followed by a *post hoc* Bonferroni test for analyses between more than two groups or within groups over time. All values are expressed as means ± SEM.

The hormone leptin is known to play a critical role regulating the immune response in sepsis [20,29]. Additionally, the physiological action of ghrelin has been observed to act reciprocally to leptin during immune activation [21]. Therefore, we asked whether additional ghrelin treatment in sepsis might affect leptin levels. To detect any influences of ghrelin on leptin levels, we measured leptin levels before and after CLP-induced sepsis. In the control group, serum leptin showed only a slight increase at the 6h time point in serum (4.7 ± 0.4 ng/ml) staying significantly lower (p = 0.029) as compared to the hfd group (Fig 3A and 3B). At later time points, serum leptin levels decreased again in controls reaching starting levels again. The hfd group showed significantly higher serum leptin levels before CLP as compared to the control group (4.6 ± 0.3 ng/ml vs. 2.7 ± 0.2 ng/ml, p = 0.001). Six hours after CLP there was a significant increase of serum leptin levels (12.9 ± 3.5 ng/ml, p = 0.029) levels in the hfd group. After 24h and 48h these levels decreased again (6.4 ± 1.9 ng/ml and 5.2 ± 1.0 ng/ml) but were still higher as compared to the starting levels and significantly higher as compared to the control group (p = 0.011 and p = 0.018). Surprisingly, ghrelin treatment increased serum leptin levels after CLP in both hfd and controls reaching significantly higher levels (p < 0.02) in the controls at all three time points as compared to the untreated controls and significantly higher serum levels in the hfd group at the 24h (p = 0.008) and 48h timepoint (p = 0.009) as compared to the untreated hfd group. Interestingly, the fold increases of leptin after ghrelin treatment was higher in the obese compared to lean mice.

IL-1ß is mainly produced by activated macrophages and stimulates thymocyte proliferation and release of prostaglandin. Interestingly, the hfd group had significantly higher baseline serum IL-1ß levels (25.5 ± 2.0 ng/ml, p = 0.026) as compared to the controls (18.1 ± 1.8 ng/ml) (Fig 3C and 3D). Sepsis induced an early increase of IL-1ß levels in both groups (42.4 ± 6.3 ng/ml and 53.9 ± 4.2 ng/ml, p = 0.016) and levels remained increased at the later time points. Ghrelin treatment completely abolished these sepsis-induced peaks in both groups keeping IL-1ß at baseline levels (6h and 24h time points, p < 0.05) or even below baseline (48h timepoint, p < 0.002).

TNF-α is an important mediator of the systemic proinflammatory host response to sepsis and is produced mainly by monocytes and macrophages. However, higher levels of this cytokines are linked to organ damage and cell necrosis [30]. TNF-α serum levels of untreated controls increased after CLP reaching significantly higher levels 48h after CLP (6.6 ± 1.4 pg/ml, p = 0.003) as compared to the 24h time point (1.5 ± 0.2 pg/ml) and the hfd group at the 48h time point (2.5 ± 0.9 pg/ml, p = 0.036) (Fig 3E and 3F). Ghrelin treatment led to significant increases of serum TNF-α levels in both groups 6 hours after CLP and stayed at these increased levels at the 48h time point (8.1 ± 2.5 pg/ml, p = 0.016 and 7.7 ± 1.3 pg/ml, p = 0.0008) and 24h (6.1 ± 1.0 pg/ml, p = 0.011 and 6.8 ± 1.1 pg/ml, p = 0.003).

### Influence of ghrelin treatment on bacterial load and cellular immune response

**Fig 4:**
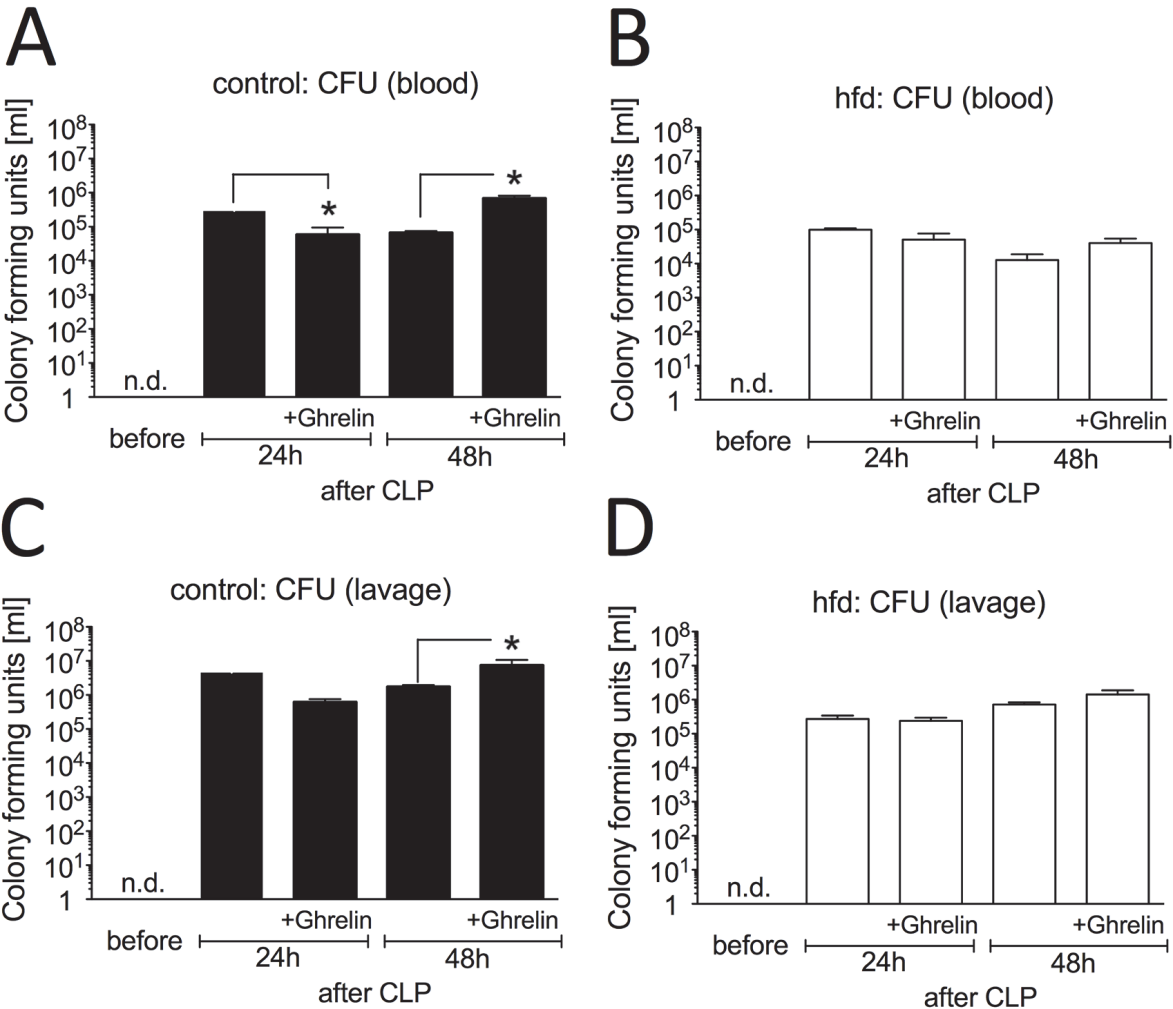
Influence of ghrelin treatment (0.5 μg/g BW, twice a day, intraperitoneal (ip.)) on bacterial load after cecal ligation and puncture (CLP)-induced sepsis. Blood and peritoneal lavage fluid from control mice (ghrelin: n = 5/timepoint, untreated: n = 5/timepoint) and high fat diet (hfd) mice (ghrelin: n = 5/timepoint, untreated: n = 5/timepoint) were collected before, 24h and 48h following CLP. Blood of (A) control and (B) hfd mice and and peritoneal lavage fluid of (C) control and (D) hfd mice were plated on tryptic soy agar pour plates and incubated at 37°C for 48 hours. Bacterial colony forming units (CFU) were determined. All values are expressed as means ± SEM; * p < 0.05 of of two-way ANOVA for unmatched samples followed by a *post hoc* Bonferroni test for analyses between more than two groups or within groups over time. n.d. = not detected.

As body temperature is essential for optimal antimicrobial host defense, we next determined whether hypothermia seen in control mice with or without ghrelin treatment had impaired bacterial clearance. Colony forming unit (CFU) counts of viable bacteria were measured in blood and peritoneal lavage fluid 24h and 48h after CLP (Fig 4). Bacterial counts of the control group were significantly higher in both blood (p = 0.042) and peritoneal lavage fluid (p < 0.032) as compared to the hfd mice. CFU levels of both hfd and control group decreased in blood and peritoneal lavage at the 48h time point with still a significant difference between the hfd and the controls (p < 0.05). Interestingly, ghrelin treatment led to an early reduction of bacterial load in the control group in both blood (p = 0.016) and peritoneal lavage (p = 0.008) at the 24h timepoint down to levels similar to the hfd group which did not show further ghrelin dependent changes at this timepoint. In contrast, further ghrelin treatment resulted in a significant increase of CFUs in blood (p = 0.005) and peritoneal lavage (p = 0.048) of the control group 48 hours after CLP reaching significantly higher levels as compared to the hfd group (p < 0.01). In the hfd group, CFU counts did not change significantly due to ghrelin treatment at the 48h timepoint. Altogether, obese mice had a decreased bacterial burden and ghrelin treatment did not significantly alter the bacterial load in contrast to lean septic mice.

**Fig 5:**
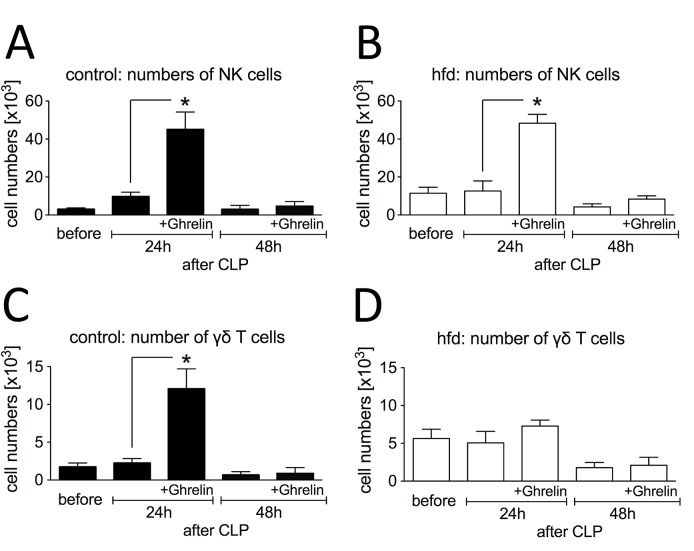
Influence of ghrelin treatment (0.5 μg/g BW, twice a day, intraperitoneal (ip.)) on peritoneal cellular immune response after cecal ligation and puncture (CLP)-induced sepsis. Peritoneal lavage fluid from control mice (ghrelin: n = 5/timepoint, untreated: n = 5–11/timepoint) and high fat diet (hfd) mice (ghrelin: n = 5/timepoint, untreated: n = 4–10/timepoint) were collected before, 24h and 48h following CLP. Numbers of neutrophils of control (A) and (B) hfd mice, natural killer (NK) cell numbers of (C) control and (D) hfd mice and γδ T cell numbers of (E) control and (F) hfd mice were were enumerated by flow cytometric analysis (combined data from two independent experiments). All values are expressed as means ± SEM; * p < 0.05 of unpaired t-test for comparison of baseline levels between two groups (hfd and control) and of two-way ANOVA for unmatched samples followed by a *post hoc* Bonferroni test for analyses between more than two groups or within groups over time.

We next investigated the effect of ghrelin on recruitment of neutrophils, NK cells and γδ T cells to the site of infection. No neutrophils were detectable in both groups before inducing sepsis (Fig 5A and 5B). Hfd mice showed a significantly higher amount of neutrophils 24h after induction of sepsis (5.4 ± 1.0 x10^3^/ml, p = 0.021) as compred to the controls (0.8 ± 0.3 x10^3^/ml). Numbers of neutrophils did not significantly change within 48 hours of inducing sepsis. Ghrelin treatment only increased neutrophil numbers in the lean cohort at the 24h timepoint (2.2 ± 0.3 x10^3^/ml, p = 0.016). After 48 hours, neutrophil numbers decreased in both hfd (3.0 ± 2.2 x10^3^/ml vs. 1.5 ± 0.2 x10^3^/ml) and controls (1.3 ± 0.5 x10^3^/ml vs. 0.5 ± 0.2 x10^3^/ml) due to ghrelin treatment.

Hfd mice also showed a significantly higher amount of NK cells in peritoneal lavage before induction of sepsis (11.4 ± 3.2 x10^6^/ml, p = 0.023) as compared to the controls (3.0 ± 0.7 x10^6^/ml) (Fig 5C and 5D). Numbers of peritoneal NK-cells did not significantly change within 48 hours of inducing sepsis. Strikingly, ghrelin treatment increased NK cell numbers in both groups significantly at the 24h timepoint (hfd: 48.4 ± 4.7 x10^6^/ml, p = 0.008 and lean: 45.0 ± 9.2 x10^6^/ml; n = 5, p = 0.002).

Similar to NK cells, the hfd group had more γδ T cells before sepsis (5.6 ± 1.2 x10^6^/ml, p = 0.04) as compared to the control group (1.7 ± 0.5 x10^6^/ml)(Fig 5E and 5F). Numbers of peritoneal γδ T cells did not significantly change within 48 hours of inducing sepsis. Ghrelin treatment only increased γδ T cell numbers in the lean cohort at the 24h timepoint (12.1 ± 2.6 x10^6^/ml, p = 0.002). Thus, ghrelin treatment resulted in increased NK cells in both lean and obese mice, while increasing neutrophils and γδ T cells in lean septic mice.

### Influence of ghrelin on neutrophil activity

**Fig 6:**
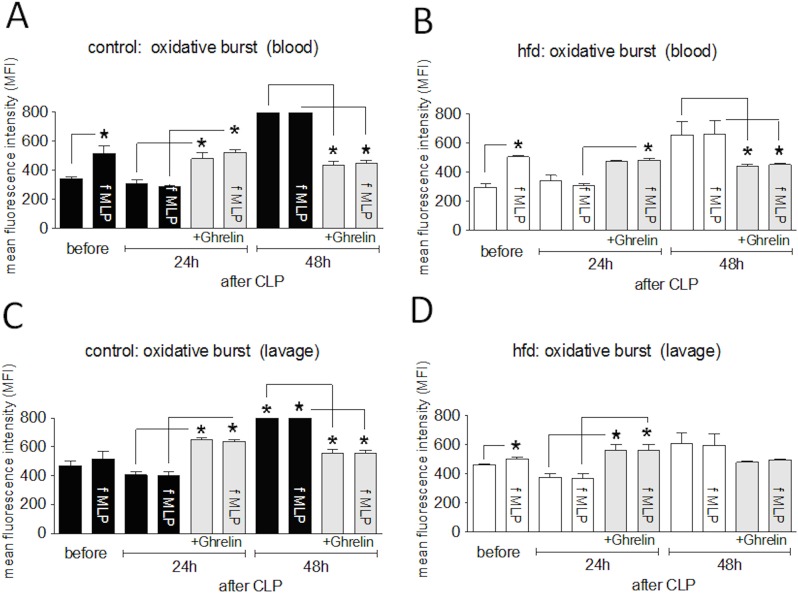
Influence of ghrelin treatment (0.5 μg/g BW, twice a day, intraperitoneal (ip.)) on oxidative burst capacity of neutrophils after cecal ligation and puncture (CLP)-induced sepsis. Blood and peritoneal lavage were collected from controls (n = 5/timepoint), controls with ghrelin treatment (n = 5/timepoint), high fat diet (hfd) (n = 5/timepoint) and hfd with ghrelin treatment (n = 5/timepoint) before, 24h and 48h after CLP-induced sepsis. Ghrelin dependent changes of spontaneous oxidative burst capacity of neutrophils and capacity after stimulation with fMLP in serum of (A) control and (B) hfd mice and in peritoneal lavage fluid of (C) control and (D) hfd mice were measured in blood and peritoneal lavage by flow cytometric analysis (combined data from two independent experiments). All values are expressed as means ± SEM; * p < 0.05 of of two-way ANOVA for unmatched samples followed by a *post hoc* Bonferroni test for analyses between more than two groups or within groups over time.

Lastly, we examined whether ghrelin treatment influences neutrophil function during sepsis by measuring oxidative burst. Spontaneous oxidative burst activity of peripheral neutrophils from untreated hfd and control mice (baseline levels before induction of sepsis) could be amplified by fMLP stimulation in both blood and peritoneal lavage fluid (Fig 6A and 6B). After inducing sepsis spontaneous oxidative burst activity of peripheral neutrophils isolated from hfd mice and control mice did not change in both groups 24h after CLP as compared to the baseline levels of both groups. Forty-eight hours after CLP we detected a significant increase in the septic control group (822 ± 19 MFI; p = 0.001) and hfd group (654 ± 94 MFI; p = 0.001) compared to baseline. Neutrophils isolated from the peritoneal lavage fluid of both groups showed a similar pattern for increased spontaneous oxidative burst activity at 24 and 48 hours after inducing sepsis (Fig 6C and 6D). In contrast to the effect on unseptic neutrophils before inducing sepsis fMLP treatment could not induce further increases of oxidative burst in both groups in blood and peritoneal lavage fluid. Ghrelin treatment led to a significant increase of spontaneous oxidative burst in both blood and peritoneal lavage fluid in both the lean and obese cohorts 24h after CLP (p < 0.01). In contrast, at the 48h time point, spontaneous oxidative burst of peripheral and peritoneal neutrophils decreased in lean mice treated with ghrelin. This effect of ghrelin treatment reducing neutrophil oxidative burst was only observed in peripheral neutrophils isolated from septic obese mice after 48 hours. Again, after fMLP treatment, no additional changes could be detected in either group of ghrelin treated mice at both septic timepoints possibly due to oxidative burst being at a maximum when the cells were isolated from the area of ongoing inflammation.

## Discussion

We observed that ghrelin treatment in early sepsis shows a bi-phasic action: in the early phase (first 24 hours) we found increased numbers of immune cells and improved oxidative burst of neutrophils associated with decreased bacterial burden. At the later phase of ghrelin treatment (48 hours), we observed attenuated neutrophil activity with increased pro-inflammatory cytokines and suppression of the anti-inflammatory response that was associated with increased short-term mortality. In the lean control group, hypothermia and a massive ghrelin dependent escalation of the bacterial burden was much more distinctive as compared to the obesity group as reflected by a worse outcome. In a previous study, we demonstrated that obesity improved survival due to increased leptin levels and improved cellular immunity [19,20]. Here we confirm an obesity-induced increase of leptin and improvement of immunity. Additionally, ghrelin treatment-induced increases in leptin levels might be responsible for normothermia, lower bacterial counts and better survival in the hfd group. Nevertheless, additional protective effects of increased leptin (e.g. survival, stabilization of body temperature and cytokine response) seem to be abolished by ghrelin treatment especially in the lean control group.

A number of studies have shown that ghrelin may exert a beneficial impact during infection or tissue injury. Previously, it was demonstrated by Wu et al. that levels of ghrelin in the lung were significantly reduced in sepsis and ghrelin administration improves organ blood flow and reduced lung injury within the first 20 hours after CLP [15]. Renal ischemia and reperfusion induced tissue damage was attenuated by ghrelin treatment within the first 24 hours [31]. Additionally, ghrelin levels were shown to be elevated after endotoxin administration [32] and could decrease pro-inflammatory cytokines [33]. Notably, these data were all collected within the first 24 hour and reflect a similar protective function. This effect is similar to that observed during sepsis due to leptin.

In the current study, we confirm the beneficial effects due to ghrelin treatment within the first 24 hours after CLP. However, in the following 24 hours, continued ghrelin activity seems to reverse these effects, with worsened cytokine response and diminished cellular defense against bacteremia. There is a time-dependent decrease in NK and γδ T cell numbers combined with increased bacterial burden and hypothermia resulting in narrowed survival in the later time period. Surprisingly, we detected ghrelin treatment-dependent leptin increases in both groups. Leptin deficiency increases susceptibility to endotoxic shock and sepsis [20,34]. Hyperleptinemia improves resistance to endotoxin and plasma leptin levels are found to be increased in survivors of acute sepsis [35]. Increased leptin levels in sepsis should be responsible for improved survival and immunity as seen before [19,20]. If ghrelin and leptin counteract one another under conditions of inflammation and sepsis, then additional ghrelin treatment after CLP could lead to a reactive leptin elevation in an attempt to maintain the balance between both regulatory components. Although these increased leptin levels may diminish the negative ghrelin associated effects in sepsis, as was observed in the hfd group, we suggest that ghrelin levels were increased before leptin levels resulting in a misbalance of function to the side of ghrelin. The increased leptin levels may not be able to counteract the ghrelin treatment, which diminishes the protective effects normally seen with elevated leptin and explains the altered survival and body temperature in our study.

### Ghrelin does not influence body temperature in early sepsis

Lean mice whether treated with ghrelin or not developed a profound hypothermia in sepsis. This fact was associated with increased bacterial burden and higher mortality. Hypothermia, or the failure to develop a febrile response in sepsis, is connected to increased organ dysfunction and worse outcome as seen in patients with septic shock [36]. Central ghrelin receptors provide a potential anatomical basis for the regulation of body temperature by ghrelin but with minor relevance under physiologic circumstances [37]. Central administration of ghrelin in rats decreased body temperature using the vagus nerve system [38] and ghrelin receptor null mice exhibited a higher core body temperature [39]. Here, we could not detect any influence of ghrelin on body temperature in sepsis. Conversely, leptin is known to regulate body temperature by its action in the brain using a leptin receptor-dependent mechanism [40]. Leptin deficiency induces hypothermia as shown in leptin-deficient ob/ob mice [41] and hypoleptinemic states in sepsis are connected to hypothermia and increased risk of infections. In our study, obese mice revealed a stabile body temperature in sepsis benefited by elevated basic leptin levels. Sepsis-induced leptin increases generated a stabile body temperature in sepsis while the ghrelin treatment itself did not alter body temperature. The inability of the lean mice to increase leptin in sepsis caused a state of hypoleptinemia which likely led to hypothermia as seen previously [19]. Interestingly, we could detect neither an additional ghrelin treatment dependent hypothermia nor a prevention of hypothermia due to ghrelin-induced leptin increases in lean mice. According to these data we suggest that ghrelin has no direct effect on body temperature under inflammatory conditions like sepsis and higher ghrelin-induced leptin levels might outweigh a possible decreasing effect of the administered ghrelin. Additionally, ghrelin administration might inhibit a leptin dependent stabilization of body temperature despite increased leptin levels as seen before due to leptin treatment [19]. This might reflect a counteracting function of ghrelin and leptin in sepsis.

### Ghrelin and the immune response

Many studies have shown that ghrelin can down-regulate inflammatory cytokines observed in the first 24 hours after sepsis induction. Ghrelin inhibits proliferation of splenic anti-CD3-activated murine T cells and inhibits their IL-1β, IFNγ and IL-10 cytokine mRNA expression in a dose dependent manner [42]. Ghrelin also inhibits IL-1β and TNFα production of LPS-treated macrophages within 24 hours [43]. In rats, ghrelin administration decreased inflammatory parameters in the lung 6 hours after CLP [33]. Chorny et al. showed in mice a ghrelin-dependent reduction of pro-inflammatory cytokines within 12 hours after LPS or E.coli administration [44]. Here, we also found a ghrelin dependent, systemic suppression of IL-6 levels starting within the first 6h after sepsis induction. In contrast, TNFα increased while IL-10 and IL-1ß levels were dramatically suppressed by ghrelin showing the beginning of the harmful action of ghrelin treatment in sepsis. IL-10 is the main anti-inflammatory cytokine in sepsis and thought to self-limit inflammatory processes [45]. IL-1ß stimulates the non-specific host response to infections and is required for successful antimicrobial responses but persistence of increased levels is associated with hypotension, shock and multi organ failure [46]. Later on, we could detect further ghrelin treatment-dependent decreases of Il-10 and IL-1ß and increases of TNFα. Despite a ghrelin-induced leptin elevation and the ability of leptin to generally improve cytokine response in sepsis [19,20] the increase in TNFα with simultaneous IL-10 and IL-1ß decline may be a sign of suppression of an early and effective immune response. This seems to be ghrelin related and might be responsible for worse outcomes.

Neutrophils respond quickly during infection and can decrease the pathogen loads by phagocytosis and intracellular killing. Another subgroup of leukocyte that respond during the early phases of sepsis include NK cells and γδ T cells. They secrete pro-inflammatory cytokines early in sepsis and are shown to reduce organ injury and mortality in sepsis [27]. After a short ghrelin-dependent increase of peritoneal cellular immune response within the first 24h and decreased bacterial burden associated with this response, we could not detect further supportive ghrelin dependent influences at later time points. After 48h, decreased γδ T cell and NK cell numbers were accompanied by high bacterial burden and increased mortality in the control group. According to these data, ghrelin seems to improve the cellular immune response only within a short early time period. Further continuous treatment, might obstruct the cytokine response without further beneficial effects on cellular immunity.

### Influence of obesity

As we have seen in a previous study, high fat diet-induced obesity (class 1) is associated with better outcomes in sepsis due to hyperleptinemia [19]. Additionally, it has been shown that ghrelin gene expression is decreased by a high fat diet [47]. Here, we observed better survival and reduced bacterial load in the septic hfd cohort. A possible explanation for these effects might be reduced ghrelin levels due to obesity that cannot be counteracted by ghrelin treatment. The elevated baseline leptin levels, combined with an improved cellular immune system and additional increases of leptin in sepsis induced by both obesity and ghrelin treatment, might tip the ghrelin—leptin balance towards leptin. This effect might be more distinct in the obese group as reflected by additional leptin-dependent normothermia compared to the control group that had lower baseline and sepsis induced leptin levels. The differences in circulating ghrelin and leptin levels in sepsis likely leads to altered immune regulation. A leptin predominance might result in an improved immune response, normothermia and better outcome. In the lean group ghrelin treatment countered leptin action and therefore reduced possible protective leptin-induced effects. This would confirm the suggestion of the existence of a reciprocal regulatory network by which ghrelin and leptin control immune cell activation and inflammation [21].
